
JOURNAL INFORMATION
==============================
NLM Title Abbreviation: Wirtschaftsdienst
Journal ID: Wirtschaftsdienst
NLM Journal ID: 101086612

Wirtschaftsdienst (Hamburg, Germany : 1949)

ISSN: 0043-6275

ARTICLE INFORMATION
==============================
PMCID: PMC7560780
PMID: 33082607
DOI: 10.1007/s10273-020-2746-8
Article ID: 2746
Article version: 1
Subjects: Ökonomische Trends

Anatomie der Corona-Krise in Europa

Konjunkturschlaglicht

Anatomy of the corona crisis in Europe

Stolzenburg Ulrich ulrich.stolzenburg@ifw-kiel.de

grid.462465.7 0000 0004 0493 2817 Institut für Weltwirtschaft, Kiellinie 66, 24105 Kiel, Deutschland
Electronic publication date: 2020 Sep 25
Print publication date: 2020
Volume: 100
Issue: 9
First page: 727
Last page: 728
Copyright: © Der/die Autor(en) 2020
License: Dieser Artikel wird unter der Creative Commons Namensnennung 4.0 International Lizenz (https://creativecommons.org/licenses/by/4.0/deed.de) veröffentlicht.
Open Access wird durch die ZBW — Leibniz-Informationszentrum Wirtschaft gefördert.
License URL: https://creativecommons.org/licenses/by/4.0/

issue-copyright-statement: © The Author(s) 2020

==============================

Amtliche Daten zu den Volkswirtschaftlichen Gesamtrechnungen liegen inzwischen für die ersten zwei Quartale 2020 vor. Sie ermöglichen eine international vergleichende Analyse der Auswirkungen der Corona-Krise auf makroökonomische Kenngrößen. Im Folgenden werden jeweils Veränderungsraten der preis-, kalender- und saisonbereinigten makroökonomischen Aggregate zwischen dem vierten Quartal 2019 (Vorkrisenniveau) und dem zweiten Quartal 2020 (Tiefpunkt der Krise auf Quartalsebene) ausgewiesen, sowohl für die Verwendungs- als auch für die Entstehungsseite. Betrachtet werden die fünf größten Volkswirtschaften des Euroraums (Deutschland, Frankreich, Italien, Spanien, Niederlande) und Großbritannien.

Die wirtschaftliche Aktivität hat sich in den Ländern sehr unterschiedlich stark abgeschwächt (vgl. Abbildung 1). So ist das Bruttoinlandsprodukt (BIP) in den Niederlanden im ersten Halbjahr „nur" um knapp 10 % zurückgegangen, und in Deutschland um 11,5 %, während die Rückgänge in Italien (17,6 %), Frankreich (18,9 %), Großbritannien (22,1 %) und Spanien (22,7 %) weitaus drastischer waren. Maßgeblich für diese massiven Unterschiede ist neben dem variierenden Infektionsgeschehen der Grad der seuchenpolitischen Eingriffe (Härte und Dauer des Lockdowns) und die jeweilige Wirtschaftsstruktur (z. B. Tourismus- oder Exportabhängigkeit).

Der Private Verbrauch ist dabei im Allgemeinen geringfügig mehr zurückgegangen als das BIP. Auffälligkeiten gibt es allerdings beim Staatsverbrauch, der vielerorts in etwa auf dem Vorkrisenniveau verblieb, nicht jedoch in Frankreich und Großbritannien, wo zweistellige prozentuale Rückgänge ausgewiesen wurden. Gleichzeitig gingen diese Rückgänge mit hohen Zuwächsen des jeweiligen Deflators einher (Frankreich 10,6 %, Großbritannien 35 %), während die Preisentwicklung des Staatsverbrauchs in den übrigen Ländern eher unauffällig war. Dies deutet auf unterschiedliche Bewertungen krisenbedingter Vorgänge durch die statistischen Ämter hin. Eine detailliertere Betrachtung zeigt, dass die Diskrepanzen durch die Konsumausgaben des Staates für den Individualbereich – also etwa Ausgaben für Bildung und Gesundheit – verursacht wurden. Konkret wurde etwa der Unterrichtsausfall an britischen Schulen als substanzieller Rückgang der Aktivität verbucht,[1] während anderswo die stetige Kostenentwicklung z. B. für Lehrergehälter das ausgewiesene, tendenziell gleichbleibende Aktivitätsniveau bestimmt. Fraglich ist, ob die Makrodaten international noch vergleichbar sind, wenn man diese hohen Diskrepanzen beim Deflator des Staatskonsums berücksichtigt. Der starke Rückgang des BIP in Großbritannien um 20,4 % im zweiten Quartal 2020 ist zum Teil auch durch diesen Effekt bedingt und lässt einen umso stärkeren Rückprall-Effekt für das zweite Halbjahr erwarten.

Der Außenhandel ist überproportional von der Krise betroffen. Insbesondere gilt dies für die Exporte aus Deutschland, Frankreich, Italien und Spanien. In Italien und Spanien sind insbesondere die Dienstleistungsexporte (Tourismus) um 55 % bzw. 62 % eingebrochen, in den übrigen Ländern erheblich weniger. In Deutschland sind zudem die Warenexporte um rund ein Viertel zurückgegangen, also weitaus stärker als das BIP, in Frankreich, Italien und Spanien liegt der Rückgang in einer Größenordnung von 30 %. Die Importe sind ebenfalls weitaus stärker zurückgegangen als

1 Zur Messung des realen Produktionsniveaus im Bildungsbereich berechnet das nationale Statistikamt in Großbritannien (ONS) gewichtete Aktivitätsindizes je nach Anwesenheit der Schüler (Wales, 2020).

Abbildung 1
**Bruttoinlandsprodukt (BIP) und Verwendungsaggregate**
Quartalsdaten, preis-, kalender- und saisonbereinigt, Veränderung zwischen viertem Quartal 2019 und zweitem Quartal 2020 in %

Quellen: Eurostat, Volkswirtschaftliche Gesamtrechnungen; eigene Berechnungen.

Abbildung 2
**Bruttowertschöpfung nach Wirtschaftsbereichen**
Quartalsdaten, preis-, kalender- und saisonbereinigt, Veränderung zwischen viertem Quartal 2019 und zweitem Quartal 2020 in %

Quellen: Eurostat, Volkswirtschaftliche Gesamtrechnungen; eigene Berechnungen.

die Wirtschaftsleistung. Überproportionale Rückgänge um mehr als 20 % verzeichneten auch die Bruttoanlageinvestitionen in Frankreich, Italien, Spanien und Großbritannien. Hierin zeigt sich vor allem die – anders als in Deutschland und den Niederlanden – weitgehende Stilllegung des Bausektors in diesen Ländern während des Lockdowns.

Dies spiegelt sich auch in der entstehungsseitigen Betrachtung wider (vgl. Abbildung 2). Die Bruttowertschöpfung im Baugewerbe ist in allen vier Ländern um rund 30 % zurückgegangen, während sie in Deutschland und den Niederlanden kaum verändert war. Die Wertschöpfung im Produzierenden Gewerbe ohne Bau, das vor allem das für Deutschland sehr bedeutsame Verarbeitende Gewerbe umfasst, ist in einer Größenordnung von 20 % eingebrochen (Ausnahme: Niederlande). Erwartungsgemäß besonders stark von der Krise betroffen sind die Wirtschaftsbereiche „Handel, Verkehr und Gastwirtschaft“ – mit nicht unerheblichen Unterschieden zwischen den Ländern – sowie „Sonstige Dienstleister“, die den Kultur- und Eventbereich umfassen. Bedeutsame Rückgänge verzeichneten auch die Unternehmensdienstleister, während die öffentlichen Dienstleister – einschließlich Bildung, Erziehung und Gesundheit – spiegelbildlich zum Staatsverbrauch in Frankreich und Großbritannien am meisten zurückgingen. Weitaus weniger betroffen von den Corona-bedingten Einschränkungen waren offenbar die Bereiche Land- und Forstwirtschaft, Information und Kommunikation, Finanz- und Versicherungsdienstleistungen sowie Grundstücks- und Wohnungswesen. Die relative Betroffenheit der Wirtschaftsbereiche für Deutschland deckt sind im Übrigen recht gut mit den Erwartungen der an der Gemeinschaftsdiagnose beteiligen Institute aus dem Frühjahr (Projektgruppe Gemeinschaftsdiagnose, 2020).

Derzeit ist ungewiss, wie lange und wie umfassend die wirtschaftliche Aktivität in den einzelnen Wirtschaftsbereichen noch behindert wird – sei es durch behördliche Vorgaben, durch individuelle Verhaltensanpassungen zur Vermeidung von Ansteckungsrisiken oder durch Auswirkungen der weltwirtschaftlichen Krisensituation über den Handelskanal. In jedem Fall ist zu erwarten, dass die Bereiche „Handel, Verkehr und Gastwirtschaft“, die Unternehmensdienstleister sowie die sonstigen Dienstleister auch in den bevorstehenden Quartalen mit direkten Einschränkungen umgehen müssen und ihre Vorkrisenniveaus kaum erreichen werden. Dies gilt in verstärktem Maße für Spanien und Frankreich, wo die Infektionszahlen zuletzt bedenklich gestiegen sind. Doch angesichts der makroökonomischen Rückkopplungen dürfte auch die Industrie – in Deutschland ebenso wie in anderen Ländern – noch einige Zeit von der weltweiten Wirtschaftskrise betroffen sein.

Ulrich Stolzenburg
Ulrich.Stolzenburg@ifw-kiel.de


Literatur

Projektgruppe Gemeinschaftsdiagnose (2020), Wirtschaft unter Schock — Finanzpolitik hält dagegen, Gemeinschaftsdiagnose im Frühjahr 2020, 43, Tab 2.10.
Wales, P. (2020), Coronavirus and the impact on measures of UK government education output, Office for National Statistics, 13. Mai, https://www.ons.gov.uk/economy/grossdomesticproductgdp/articles/coronavirusandtheimpactonmeasuresofukgovernmenteducationoutput/2020-05-13 (11. September 2020).
